# Bulk-Like SnO_2_-Fe_2_O_3_@Carbon Composite as a High-Performance Anode for Lithium Ion Batteries

**DOI:** 10.3390/nano10020249

**Published:** 2020-01-30

**Authors:** Jie Deng, Yu Dai, Zhe Xiao, Shuang Song, Hui Dai, Luming Li, Jing Li

**Affiliations:** 1 College of Pharmacy and Biological Engineering, Chengdu University, Chengdu 610106, China; dengjie@cdu.edu.cn; 2 Department of Chemical Engineering, Sichuan University, Chengdu 610065, China; daiyuscu@163.com (Y.D.); 2016323050027@stu.scu.edu.cn (S.S.); daihui18@cdut.edu.cn (H.D.); 3 Institute of New Energy and Low Carbon Technology, Sichuan University, Chengdu 610207, China; 2017226220007@stu.scu.edu.cn; 4 College of Materials and Chemistry & Chemical Engineering, Chengdu University of Technology, Chengdu 610065, China; 5 Institute of Advanced Study, Chengdu University, Chengdu 610106, China

**Keywords:** lithium ion battery, tin oxide, anode, stress management, carbon, high rate handling

## Abstract

Boosted power handling and the reduced charging duration of Li ion cells critically rests with ionic/electronic mobility. Ion mobility in electrochemically relevant grains normally stands for an essential restriction of the velocity at which the energy of a cell can be stored/released. To offset sluggish solid-state ionic transport and achieve a superior power/energy density rating, electroactive grains often need exquisite nanoscaling, harming crucial virtues on volumetric packing density, tractability, sustainability, durability, and cost. Unlike elaborate nanostructuring, here we describe that a SnO_2_-Fe_2_O_3_@carbon composite—which adopts a metal oxide particles-intercalated bulk-like configuration—can insert many Li^+^ ions at elevated speeds, despite its micro-dimensionality. Analysis of charge transport kinetics in this tailor-made architecture unveils both much improved ion travel through compact monolithic substances and the greatly enhanced ion access to surfaces of SnO_2_/Fe_2_O_3_ grains. According to the well-studied battery degradation mechanism, it is that both the effective stress management and internal electric field in our as-prepared sample that result in recommendable capacity, rate behavior, and cyclic lifespan (exhibiting a high reversible capacity of 927 mAh g^−1^ at 0.2 A g^−1^ with a capacity retention of 95.1% after 100 cycles and an ultra-stable capacity of 429 mAh g^−1^ even over 1800 cycles at 3 A g^−1^). Unique materials and working rationale which ensure the swift (de)lithiation of such micrometer-dimensional monoliths may open a door for various high-power/density usages.

## 1. Introduction

With the technical progress in recent years, lithium ion batteries (LIBs) have captured growing attention as promising candidates for traditional energy storage devices, because of characteristics of high energy efficiency, wide temperature adaptation range, long cyclic life, eco-benignancy, no memory effect, light weight, rapid charging/discharging dynamics (due to the minimum metal ion diameter relative to other metal ions), small volume, and low self-discharge. Because the ion lithium storage performance inherently depends on the electrode materials, the novel excellent-performance electrode substances which manage to deliver a great deal of energy for several minutes instead of hours are in high demand, to heighten the power output and reduce the charging duration within LIBs. These substances can hence expedite the technical leaps relevant to the deployment of portable/flexible electronics industries, vehicular sectors, and smart grid systems, and meet a massive demand for a novel large power-consuming energy storage system. The most generally applied routes for enhancing the energy/power rating properties can deal with the introduction of myriads of nanoscaled or/and holey (usually graded) architectures, which can—to the largest degree—shorten the Li^+^ solid-like movement lengths, permit the faster lithium passage across whole electrodes, and enlarge the accessible interface boundaries between electrode substances and electrolytes. Besides, lots of intricate morphological engineering avenues involving the nanosheets, nanowires, nanotubes, hollow/yolk-shell, specific crystal facet exposure, and nanocubes have been widely used in conjunction with the powerful but sophisticated surface coating (SiOC, carbon, Si, TiO_2_ and so on). These strategies aim at guaranteeing the effective stress relaxation of electrochemically active phases during lithiation, which can generally have a significant impact upon addressing the variables such as the electronic conductivity, hysteresis problem between discharge and charge (intrinsic to conversion reactions), reversible capacity retention and working voltage during cycling, another several key points of the usage of large current loads [1,2,3,4,5].

Actually, notwithstanding the superb lithium migration, graphite will dysfunction under large charge/discharge speeds as a result of adverse stress intensification and lithium dendrite growth, the latter inducing some safety issues like short circuiting, fire, and explosions [6,7,8,9,10]. Such fatal drawbacks can intrinsically stunt the wide employment of the low-potential negative electrodes for high-rate usage, in that the electrode nonuniformity or any source of the augmented overpotentials will trigger off the harmful lithium galvanization potentials on the electrode surface or/and serious surface cracking/disintegration [6,7]. Tetragonal rutile-typed SnO_2_ nanocrystallites with a mean working potential (0.6 V vs. Li^+^/Li), possess the great-theoretic capacity and big-rate conversion-(de)alloying chemistry under no threat of a lithium dendrite production or marked solid-electrolyte interface evolution. However, SnO_2_ particles inevitably undergo a detrimental decay of the full-cell potential and thus the energy content [11,12,13,14]. For this sufficiently-identified “elevated” potential and high-rate negative electrode, a reversible Li^+^ storage capacity of the highly graded flower-like micro-spheres of ~2 μm assembled by thin SnO_2_ nanosheets as subunits approaches roughly 400 mAh g^−1^ under a high current density of 800 mA g^−1^ [11]. In comparison, over long-term study, hybridization with various carbonaceous allotropes has invested SnO_2_ with a far superior reversible capacity—even reaching 634 mAh g^−1^ at 6.4 A g^−1^—equivalent to about 3.7 lithium ions per transition metal (Li^+^/TM) (TM = transition metal) [11]. Nonetheless, the application of nanoscaled and hierarchically holey structures in electrochemical energy storage technologies innately suffers from acute prejudice of volumetric and areal energy density owing to ultralow packing density. Besides, fabricating and characterizing such exquisitely engineered nanostructures might hardly evade both the supramolecular chemistry and in situ monitoring tools, always resulting in the high cumbersomeness and cost. The pertinent preparation protocols rarely get rid of both small product throughput or/and concomitant formation of chemical wastes, whilst also encountering performance deterioration in the course of repeated charging/discharging (due to events like the catalytic dissociation of electrolytes and topological fluctuation) [15,16,17,18]. Simply speaking, the active anode materials with poor electrical conductivity and structural stability—as well as low Li-ion diffusivity and transition-metal cation dissolution—can markedly degrade the performance (capacity, rate performance, and cycling stability) of LIBs. In particular, the morphological evolution of such electrode materials can severely abolish the nano-structuring and exacerbate the initial capacity fading.

Herein, we abandon the traditional morphological protocols to construct and downscale the electroactive substances at the nanometre level for coping with the inferior ion mobility and electron conductivities (particularly occurring to the SnO_2_ oxides). We exhibit that, using a suitable host skeleton, any of the routine architecture, dimension, or porousness tenets can be no longer in high demand for enabling the pragmatic high-rate electrochemical materials. Contrastingly, in light of the prior researches into the complex binary SnO_2_-based metal composite—for instance, the SnO_2_-Fe_2_O_3_ hybrid system [19,20]—and into robust/fast ion-transmitting hosts like the connective carbon substrate framework [21]; the structural motifs are corroborated that ought to feature the conducive lithium ion motion function and, hence, the excellent electrochemical signatures, qualifying the micro-dimensional compact granules for the exceedingly large rates. We discover that, as long as the ultra-small interstitial-devoid 3d metal oxide nano-particulates conjoin with a proper carbon host lattice, the very large reversible capacity and rates can be attained. The dense, bulk-like composite monolith studied here typifies a series of complex metal oxide-intercalated carbon architecture, primarily with the ultrafine SnO_2_/Fe_3_O_4_ nanocrystals being homogenously implanted among carbon layers in a micron dimensional elastic/conductive protecting carbon sheath. More importantly, this compound can be easily derived from a scalable and facile gels pyrolysis scenario. Briefly, the xerogels of the metal ion-uniformly complexed chelates generated by concentrating/polymerizing the clear gel of citrate and metal salts were thermally carbonized under N_2_ at 600 °C to create the compact metal-embedded carbon monolith that is adept at conducting the electrons and lowering the stress during electrochemical actions. Its distinctive lithium ion storage properties can be preliminarily pinpointed via scrutinizing the outsized (10~20 μm) compact grains of a metal oxide nanoclusters-inside-carbon configuration (B-SFO@C). Albeit pursuant to the weight-normalized benchmarking, the electro-chemical behaviour of B-SFO@C can notably exceed that of the nanoarchitectured congeners of the tremendously probed SnO_2_ and SnO_2_/carbon hybrids under analogous weight loading circumstances. Seeing that the large population of metal oxides and the great tap density of our monolithic B-SFO@C is in significant contrast with the extensively reported nanostructures, it will result in a rather impressive volumetric behaviour, heralding huge potential towards the practical feasibility. On the basis of widely recognized battery degradation and working mechanism, the well-preserved morphological integrity and the superior cycling stability—in combination with the high reversible capacity—intrinsically reflects that this unique secondary structure with inactive/active buffers via the bulk-like binary metal oxide-intercalated carbon composite can effectively create the built-in potential (electric field) around hetero-interfaces and manage the stress generation through relaxation which might be intensified to the electrode material surface. Establishment of structural and morphological integrity through introduction of a stress management structure can result in superior Li ion storage functions. In short, given both the tractability and reproducibility of our synthetic methodology, we depict the generality of such micro-dimensional or bulk effect via studying one sort of alternative anode substances, i.e., the compact binary metal nanocrystals-embedded carbon configuration. The confinement of mixed metals into a carbon monolith results in merging of the intrinsic properties of individual components at the nanoscale in a manner that ushers in new advanced materials for the high-rate energy conversion and storage devices. 

## 2. Materials and Methods

### 2.1. Materials Synthesis

**Figure 1:**
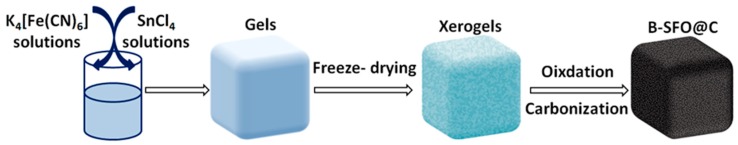
Schematic formation processes of B-SFO@C.

The schematic formation process of the materials is illustrated in Figure 1. A typical procedure to synthesize the B-SFO@C sample is as follows: 0.2 M SnCl_4_ aqueous solution containing 0.1 g mL^−1^ of citric acid and 0.2 M K_4_[Fe(CN)_6_] aqueous solution containing 0.1 g mL^−1^ of citric acid was simply mixed with a volume ratio of 3:1 at room temperature and a clear gel was obtained after a few minutes. Then, the as-prepared gel was freeze-dried overnight. Subsequently, the obtained xerogels were firstly pyrolyzed at 400 °C in air for 2 h, and then carbonized at 600 °C in N_2_ for 2 h. The resulting product was washed with the distilled water and ethanol to remove the impurities and dried at 100 °C in air. The final products were designated as B-SFO@C. B-SFO was prepared by the similar procedure except for replacing N_2_ with air. B-SO@C was prepared by removing the Fe_2_O_3_ in B-SFO@C by etching with a 0.5 M oxalic acid solution. Simply, the selective leaching was achieved by dispersion of the B-SFO@C products into a 0.5 M oxalic acid aqueous solution by ultrasonication for 30 min and then the robust agitation overnight. The pure carbon framework was derived by following procedures. The B-SFO@C is dispersed into 2 M NaOH aqueous solution by ultrasonication for 30 min. After that, the above suspension was transferred into a 100 mL Teflon-lined stainless-steel autoclave and subjected to hydrothermal treatment at 180 °C for 24 h to remove the SnO_2_. Subsequently, the obtained products were dispersed into 0.5 M oxalic acid aqueous solution by ultrasonication for 30 min and then stirred over night to remove Fe_2_O_3_. The resulting products was collected by centrifugation, washed three times with DI water and ethanol, and then dried in an oven at 100 °C to get the carbon matrix.

### 2.2. Materials Characterization

The crystal structures of B-SFO@C were characterized by X-ray powder diffraction (XRD, DX-2700, Cu *K*_α_ radiation, *λ* = 1.542 Å). The carbon content in the B-SFO@C was examined by thermogravimetric analysis (TGA, Bulid 193). The carbon chemical bonding in B-SFO@C was analyzed by Raman spectra on a LabRAM HR Raman spectrometer (Tokyo, Japan) using laser excitation at 632.8 nm. Scanning electron microscopy (SEM, JEOL, JSM-7500F, Tokyo, Japan) and transmission electron microscopy (TEM, Tecnai G2 F20 S-Twin, FEI, Hillsboro, OR, USA) were performed to observe the microstructure of the samples. The surface electron state of B-SFO@C was identified by X-ray photoelectron spectroscope (XPS, AXIS Ultra DLD, Kratos, Manchester, UK). The nitrogen adsorption and desorption tests were performed using a NOVA 1000e analyzer (Quantachrome Instruments, Boynton Beach, FL, USA).

### 2.3. Electrochemical Analysis

The working electrode was prepared by mixing the active materials, polyvinylidene fluoride binder, and conducting carbon (acetylene black, Sigma-Aldrich, St. Louis, MO, USA) in *N*-methyl-2-pyrrolidinone to form homogeneous slurry with a weight ratio of 8:1:1. The slurry was doctor bladed on copper foil current collector and then dried in a vacuum oven for 12 h. The copper foil coated with the active materials was then stamped into the rounds with a diameter of 14 mm. Electrochemical measurements were executed using the CR-2032-type coin cells which were assembled in a glove box (O_2_ and H_2_O contents < 1 ppm) filled with highly pure argon. A pure lithium foil was used as the counter electrode, with the Celgard 2400 microporous polypropylene membrane (Celgard, 2400, 25 μm in thickness, Charlotte, NC, USA) as the separator and 1 M LiPF_6_ in ethylene carbonate (EC) and dimethyl carbonate (DMC), with a volume ratio of 1:1 as electrolytes. The galvanostatic charge/discharge tests were carried out on a Neware battery measurement system (Neware Technology Ltd., Shenzhen, China). The cyclic voltammetry (CV) measurements were implemented on a CHI 660E electrochemical workstation (Shanghai, China). The electrochemical impedance spectroscopy measurement was done with an electrochemical analyzer within a frequency range of 10^5^–0.01 Hz.

## 3. Results and Discussion

**Figure 2:**
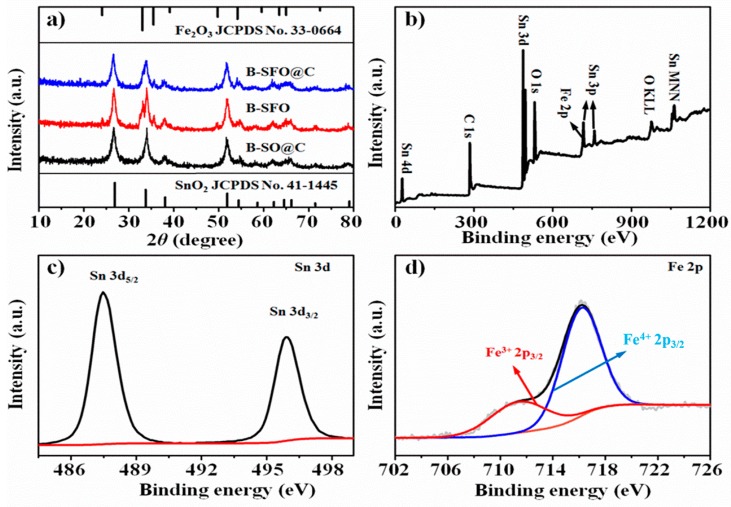
(**a**) XRD pattern of the samples, (**c**,**d**) XPS spectra of B-SFO@C sample: (**b**) survey spectrum, (**c**) Sn 3d, (**d**) Fe 2p.

**Figure 3:**
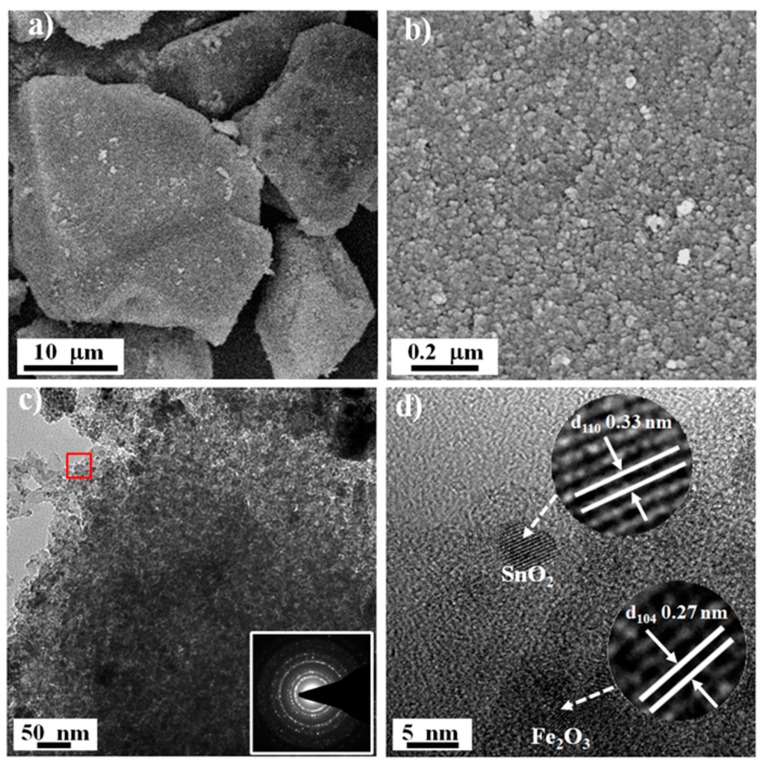
Micromorphology of B-SFO@C sample: (**a**) Low- and (**b**) High-resolution SEM image, (**c**) Low-resolution TEM image and corresponding SAED pattern (inset), (**d**) HRTEM image.

The X-ray diffraction (XRD) was conducted to substantiate the structural attribute of the samples. As shown in Figure 2a, the B-SFO@C and B-SFO samples exhibit the analogous XRD patterns. The diffraction peaks could be assigned to both the tetragonal SnO_2_ (JCPDS card No. 41-1445) and the rhombohedral Fe_2_O_3_ (JCPDS card No. 33-0664), manifesting that the successful generation of SnO_2_ and Fe_2_O_3_. In addition, the diffraction peaks indexed to Fe_2_O_3_ vanish in B-SO@C, evidencing that the Fe_2_O_3_ phase has been eliminated after etching using the oxalic acid solution. The surface electronic structure and chemical element valance state of B-SFO@C was examined by X-ray photoelectron spectroscopy (XPS). The survey spectrum (Figure 2b) corroborates existence of C, Sn, Fe, and O. Two predominant strong peaks are monitored at 487.4 and 495.9 eV, corresponding to the Sn 3d5/2 and Sn 3d3/2 for Sn^4+^ in the Sn 3d core emission (Figure 2c), respectively, which indicates the presence of Sn^4+^ in B-SFO@C [22]. Meanwhile, the XPS spectrum of Fe 2p (Figure 2d) can be deconvoluted into two envelopes at 711.3 eV and 716.3 eV. These two bands should be assigned to the Fe^3+^ 2p_3/2_ and Fe^4+^ 3p_3/2_, respectively, which implies the coexistence of Sn^4+^ and Fe^3+^ in B-SFO@C [23]. The C 1s signal (Figure S1) can be fitted into three peaks, responsible for the C–C/C=C (284.6 eV), C–O (286.0 eV) and C=O (288.6 eV) [24]. Noteworthily, the weight fractions of SnO_2_ and Fe_2_O_3_ calculated from XPS are considerably lower than those computed from the inductively coupled plasma atomic emission spectroscopy (ICP) as shown in Table S1, elucidating that most of SnO_2_ and Fe_2_O_3_ nanoparticles are located inside carbon matrix to yield a metal oxide nanoparticle-intercalated carbon structure. Such preferential in-carbon-lattice enrichment can also be validated by TEM images (Figure 3c,d). Thermogravimetry analysis (TGA) was carried out to further quantify the carbon content in B-SFO@C (Figure S2) and the final carbon content is approximately 32%. In other words, the overall metal or/and metal oxide percentage is as high as 68%, suggesting a high metal loading density. Raman spectrum of B-SFO@C (Figure S3) illuminates that the intensity ratio between D-band and G-band (I_D_/I_G_) is as high as 0.98, indicating that the carbon in B-SFO@C is amorphous [25]. The relatively low graphitization can ensue from the low pyrolysis temperature of 600 °C.

The micromorphology of B-SFO@C was determined using scanning electron microscopy (SEM) measurements (Figure 3a). Clearly, the B-SFO@C sample presents a dense bulk structure with a size ranging from 10 up to 20 μm, indicating that the xerogels frameworks were effectively stabilized and maintained during oxygen-lean pyrolysis. A close inspection of the magnified SEM image (Figure 3b) unveils that the surface of the monolithic B-SFO@C is constituted of closely packed fine particles, with only an extremely small number of pores/voids. In addition, the element mapping images (Figure S4) reveal the uniform spatial distribution of C, O, Sn, and Fe elements throughout the whole framework of B-SFO@C. The tap density of B-SFO@C measured using the tap density tester is about 0.71 g cm^−3^—higher than that of various nanosized SnO_2_-based composites (about 0.4 g cm^−3^) reported previously [26,27]. Such high tap density will become largely useful to facilitate homogenous slurry mixing and heighten the energy density and volumetric performance. The N_2_ volumetry (Figure S5) displays that the BET surface area of B-SFO@C is only 12.3 m^2^ g^−1^, which is one order of magnitude lower than a variety of the nanosized SnO_2_-based composites (>100 m^2^ g^−1^) recorded before [24,28]. Such textural feature again signifies a compact structure of B-SFO@C, in good consistency with SEM pictures (Figure 3a,b). Besides, the B-SO@C and B-SFO samples also demonstrate the similar bulk structure (Figure S6a,b). However, the surface of B-SFO (Figure S6c) is rougher than that of B-SFO@C and is made up of many particles with a size of 100–200 nm. The morphology disparity between B-SFO@C and B-SFO is due to the fact that the carbon matrix can serve as the strong spacer to effectively encumber the agglomeration, growth, and coarsening of metal oxide particles during pyrolysis. Detailed microstructures of B-SFO@C can be further analysed using transmission electron microscopy (TEM). In Figure 3c, a high density of dark spots as metal nanoparticles are closely and homogeneously distributed in the carbon matrix. The corresponding selected area electron diffraction (SAED) pattern (Figure 3c inset) shows the bright polycrystalline diffraction rings of SnO_2_ and Fe_2_O_3_ [29]. The magnified TEM image (Figure S7) further reveals that the ultra-small nanoparticles with a size of about 5 nm are evenly implanted within the interiors of the carbon framework, giving rise to a unique metal particles-in-carbon sandwich-like configuration. In addition, as shown in Figure 3d, the high-resolution TEM (HRTEM) sheds light on two sets of the well-defined crystal lattice fringes with the interplanar distances of 0.33 nm and 0.27 nm, pertinent to the (110) facet of SnO_2_ and (104) facet of Fe_2_O_3_, respectively. Besides, a clear monolithic contour with no well-observable porosity (including mesopores and macropores) occurs in the TEM images (Figure 3c,d), again proving non-porous/compact structural motifs. The vermiculate domains tightly around dark spots (metal nanoparticles) with the poorly visible lattice lines can be indexed as amorphous carbon layers, in good consistency with the noncrystalline characteristics revealed by the XRD and Raman analysis. According to the previous rationale that stress can be assuaged through downscaling towards the nanometre, a dimension threshold to cause the expansion and spread of fracture in the materials has been proven to be roughly 150 nm [30,31,32]. Aa a result, multitudinous exquisite morphological engineering toward highly hierarchical structures have been under study, to fulfil the Sn stress relaxation during the lithiation process. For instance, the marked boosts of long-cycle stability have come true by exploiting the sub-10-nm-sized Sn/SnO_2_-based nanoparticles encapsulated inside the nitrogen/phosphorus co-doped hierarchically porous carbon and reduced graphene oxides [14], a mesoporous carbon@SnO_2_@ carbon hollow nanosphere with dual shells [33], the Sn-SnO_2_ hybrid nanoclusters embedded inside carbon nanotubes [34], the heterostructured SnS-ZnS@C hollow nanoboxes embedded in the graphene [35], the carbon-coated SnO_2_–CoO yolk–shell microspheres [36], the double shell micro-cube assembled by nanosized Co_3_Sn_2_/SnO_2_ heterostructures with amorphous carbon layers wrapped inside the three-dimensional graphene matrix [37], a mixture of porous hollow SnO_2_ nanocube and graphene aerogel [38], the novel honeycomb-like composite composing of the carbon encapsulated SnO_2_ nanospheres embedded in the carbon film [39], the sandwich-like C@SnO_2_/Sn/void@C hollow spheres [40], the carambola-shaped SnO_2_ wrapped within the carbon nanotube network [41], the core-shell structured Cu_6_Sn_5_@SnO_2_-C nanocomposites [42], the chestnut-like SnO_2_/SnO_2_/C nanocomposites with the hierarchical structures [43], and the ultrafine SnO_2_ aggregates in interior of porous carbon nanotubes [44]. Disappointingly, the realistic usage of such examples has been hampered by the small tap density [26,27] of the hierarchical layouts, as a consequence of the existence of empty room/volume and other topological problems. Worse still, to secure the empty rooms will deprive the materials of vital virtues during the tight calendaring in that majority of empty volume might undergo the utter devastation. Herein, the tap density increases through constructing a compact secondary architecture featuring the inactive/active buffers by composites. Confining a high population of the ultrafine metal nanoparticles inside the bulk-like carbon monolith can not only modulate the stress but also obviate a great deal of void space and, therefore, the related morphological problems. In this sense, such a binary metal oxide nanoparticles-intercalated carbon monolith structure is concluded to be more amenable to traditional electrode preparation than other designer nanomaterials, particularly taking into account the real-world utilization based upon the high average energy/power density rating. As for LIBs, this material is projected to be distinguished by an elevated specific capacity, endurable cyclic behaviour, and oppressed volume swelling; thus, a small irreversible capacity loss and a resultant high capacity retention.

**Figure 4:**
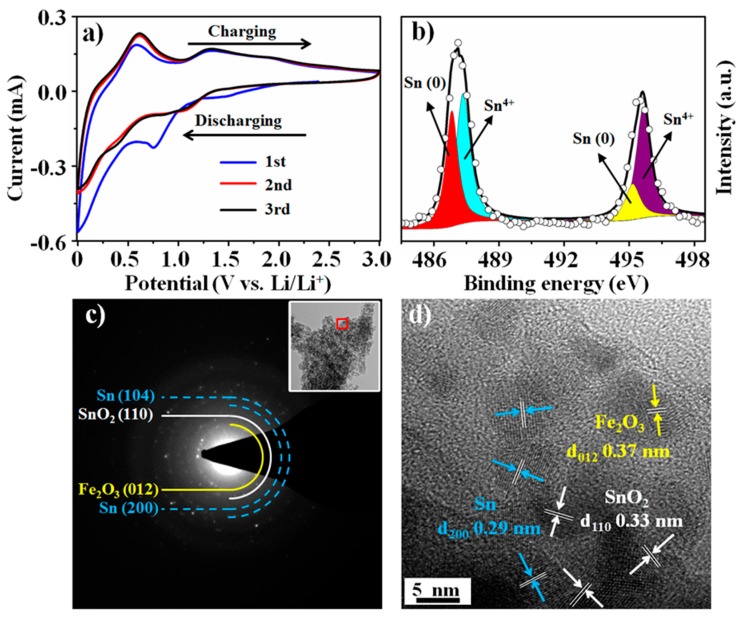
(**a**) cyclic voltammetry (CV) curves of B-SFO@C electrode at a scan rate of 0.1 mV s^−1^, (**b**) The X-ray photoelectron spectroscopy (XPS)spectrum of Sn 3d for B-SFO@C at full-charged state, (**c**) selected area electron diffraction (SAED) pattern of B-SFO@C at full-charged state and corresponding low-resolution TEM image (inset), (**d**) HRTEM image of B-SFO@C at full-charged state.

The CV curves of the B-SFO@C electrode at a scan rate of 0.1 mV s^−1^ from 0.005 up to 3.0 V were shown in Figure 4a. The reaction between B-SFO@C and lithium occurs in three domains from 0.7 to 1.41 V, with the three regions very analogous to the ones identified for other Sn-based materials, for example, SnO_2_/C, Co_3_Sn_2_/SnO_2_, and SnS-ZnS@C. Thus, the peaks emerging at 1.41 V, 0.77 V and < 0.7 V in the first cathodic scan process can deal with conversion reaction of SnO_2_ and Fe_2_O_3_, formation of SEI film and alloying process of Li^+^ inserting into Sn, respectively [45,46]. In the subsequent anodic scan process, the strong peaks at 0.59 V and the weak peak at 1.89 V originate from the dealloying event of Li*_x_*Sn to Sn and the oxidation of Fe to Fe_2_O_3_, respectively. The peak occurring around 1.3 V is assumed to be reversible conversion reaction of Sn to SnO_2_ although the conversion reaction of SnO_2_ is universally deemed irreversible [47]. To set forth the chemical valence state of Sn-based oxides at a full-charged state, the XPS was conducted (Figure 4b). The XPS spectrum of the Sn 3d core shell can be partitioned into four main peaks. The ones at 487.4 and 495.9 eV belong to the 3d_5/2_ and 3d_3/2_ for Sn^4+^, respectively. The signals at 486.7 and 495.2 eV match with the 3d_5/2_ and 3d_3/2_ for Sn(0), respectively, supporting the fact that the metallic Sn phase was indeed oxidized to SnO_2_. In addition, the SAED pattern of the full-charged B-SFO@C electrode (Figure 4c) clearly demonstrates the diffraction rings attributable to SnO_2_, Sn and Fe_2_O_3_, while the HRTEM image (Figure 4d) reveals the characteristic crystal lattice stripes of SnO_2_, Sn and Fe_2_O_3_. This further indicates the coexistence of SnO_2_ and Sn. The supra results doubtless suggest the reversible conversion reaction between Sn and SnO_2_. The most recent reports have also postulated that metallic nanoparticles such as Fe, Co and Mn can work as a trigger or catalyst to trigger off the decomposition of Li_2_O, thereby conducive to the reversible generation of SnO_2_ [48]. The different oxidation potentials of Sn to SnO_2_ and Fe to Fe_2_O_3_ in B-SFO@C allow the Fe phases produced from the conversion reaction of Fe_2_O_3_ to call into play the unique role of such triggers or catalysts. Therefore, the electrochemical reactions of the B-SFO@C electrode during cycling can be described as follows:

SnO_2_ + 4Li^+^ + 4e^−^ ↔ Sn + 2Li_2_O (1)

Sn + *x*Li^+^ + *x*e^−^ ↔ Li*_x_*Sn (0 ≤ *x* ≤ 4.4) (2)

Fe_2_O_3_ + 6Li^+^ + 6e^−^ ↔ 2Fe + 3Li_2_O (3)

**Figure 5:**
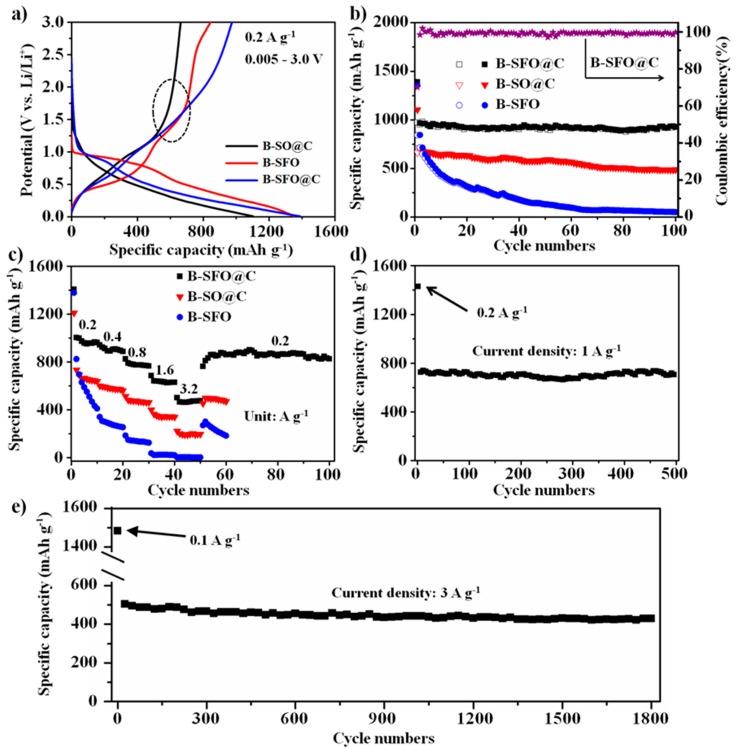
(**a**) Initial discharge/charge curves of B-SFO@C, B-SO@C and B-SFO electrodes at 0.2 A g^−1^ in the potential range of 0.005–3.0 V, (**b**) cyclic behavior at 0.2 A g^−1^ and (**c**) rate capability summary at various current densities from 0.2 A g^−1^ to 3.2 A g^−1^ of three electrodes, long-term high-rate cycling performance of the B-SFO@C electrode at (**d**) 1 A g^−1^ and (**e**) 3 A g^−1^.

Galvanostatic discharge/charge profiles of B-SFO@C, B-SO@C and B-SFO electrodes at 0.2 A g^−1^ between 0.005–3.0 V are shown in Figure 5a to probe the favourable bulk or micro-dimensional effect of the metal-intercalated carbon structure upon battery performance. In comparison to the B-SO@C electrode, the B-SFO@C electrode features a more obvious charge plateau in the range of 1.0–1.5 V, hinting that reversibility of the conversion reaction of Sn/SnO_2_ in the B-SFO@C electrode is superior to that of the B-SO@C electrode. This also reveals the vital impacts which the Fe component exerts upon manipulating the electrochemical features of SnO_2_-based materials. The initial discharge/charge capacity of B-SFO@C, B-SO@C and B-SFO electrodes reaches 1389/973, 1103/662 and 1353/842 mAh g^−1^, respectively, with corresponding initial Coulombic efficiencies (ICE) of 70%, 60% and 62%. Strikingly, the ICE of the B-SFO@C electrode here exceeds not only the values of B-SO@C and B-SFO electrodes, but also the counterparts of many elaborated nanosized SnO_2_-based composite anodes (45–55%) recorded to date [26,49,50]. The central reason behind the high ICE of the B-SFO@C electrode is that the irreversible capacity loss engendered by both the formation of SEI film and by the irreversible conversion chemistry of SnO_2_ is pronouncedly weakened. In other words, the much smaller ICE of B-SO@C and B-SFO correlate with the absence of the binary intercalation structure, which leads to the production of irreversible phases of Li_2_O and Li*_x_*Sn components in the lithiation event. Besides, the inherently poor electrical conductivity of single metal Sn phases induces the relatively unsatisfactory initial discharge/charge capacities. Such disadvantage can be addressed via introducing the second component of Fe (irrespective of carbon skeleton), which remarkably enhances both the ion access to and the electron conveyance along the electroactive materials.

The kinetics of the B-SFO@C, B-SO@C and B-SFO electrodes were evaluated over a varying range of current densities between 0.2 and 3.2 A g^−1^, and B-SFO@C features the attractive bulk rate capability in standard electrode formulations. As seen in Figure 5c, the average discharge capacities of B-SFO@C at 0.2, 0.4, and 0.8 A g^−1^ amount to 958, 890, and 774 mAh g^−1^, respectively. Thus, roughly 5.60~4.53 Li^+^/TM (TM means transition metal) can be reversibly intercalated to deliver energy, while B-SFO@C sustains a capacity of 3.71 Li^+^/TM, despite a gain in rate by a factor of eight. At 3.2 A g^−1^, which means a rate enhancement by 16 folds, it still remains probable to swap 2.70 Li^+^/TM and access 462 mAh g^−1^. For the sake of checking the rate behaviour in more rigorous environments, immediately after being assessed at 3.2 A g^−1^, the current density reverts to 0.2 A g^−1^, at which the test was cycled over 50 runs. In such circumstances, 4.46 Li^+^/TM (around 762 mAh g^−1^) can get reversibly inserted, with a 4.82 Li^+^/TM exchanged for a reactivated/settled capacity of 825 mAh g^−1^ after 40 consecutive cycles. The rate behavior in the binary metal-in-carbon configuration exceeds a variety of anode materials and even keeps abreast with some lithium solid electrolytes. The high rate capability inherently correlates with the excellent mobility/transport of lithium ions in the composite under diverse current densities. In this sense, the rate outcomes suggest that the speedy lithium ion motion can be achieved to the large lithium contents (up to 2.7 Li^+^/TM). Supposing the further optimization of this binary metal-inserted carbon structure, its intrinsic scope for the high-rate multi-redox chemistry will escalate up to a much greater level of Li^+^/TM. The finding that the micrometer-sized monolith of the binary metal-intercalated carbon composite (B-SFO@C) shows extraordinary rate behavior and could be cycled under the very elevated rates unravels the superior structural resilience, particularly given its good electrolyte infiltration and the short Li^+^/electron diffusion length. By contrast, the exceptional lithium storage properties cannot be detected for the other two composites with different chemical compositions and structural motifs. B-SO@C attains an average capacity of 663, 580, 474, 338 and 190 mAh g^−1^ at 0.2, 0.4, 0.8, 1.6 and 3.2 A g^−1^, respectively, convincingly uncovering that the iron component takes an essential part in enhancing the discharge capacities. The B-SFO electrode confers an average capacity of 588, 282 and 138 mAh g^−1^ under 0.2, 0.4 and 0.8 A g^−1^, respectively, and is even hardly discharged to store Li ions at 1.6 and 3.2 A g^−1^, which reveals a very poor rate ability. The rather similar capacity at the low current density and the increasing gulf at high current density between these electrodes justifiably unveil the primary role which the carbon skeleton plays in improving the lithium diffusion. That is to say, regardless of a single or binary metal system, the carbon matrix is in high demand for attaining the elevated capacity retention, though a small discharge capacity occurs to B-SO@C. The vivid performance comparison above univocally denotes the indispensable implications that both the carbon framework and iron phase have upon tuning the lithium ion storage process. Put another way, the intercalation of binary metals inside a carbonaceous bulk matrix can result in a good integration of the innate functions of the respective entities in the nanoscale, in a manner which produces the novel composite materials for optimizing the insertion of lithium ions at broad rate loading.

In addition, the long-term cycling performances of the B-SFO@C, B-SO@C and B-SFO electrodes were appraised at 0.2 A g^−1^ (Figure 5b). The B-SFO@C electrode can maintain a very stable and high capacity of 927 mAh g^−1^ with a sustainable retention of 95.1% even after 100 cycles, indicating an exceptional cyclic lifetime. Note that, though the ICE of B-SFO@C is relatively low, the coulombic efficiency can rapidly increase to 92% in the second cycle and can remain over 98% in subsequent runs. However, both the B-SFO and B-SO@C electrodes only give the decaying cycling performance over 100 cycles with a capacity of 51 and 488 mAh g^−1^, respectively, concomitant with a corresponding retention of merely 6% and 68.4%. The pure carbon matrix delivers a constant but low capacity of 232 mAh g^−1^ after 50 cycles (Figure S8), whereas the carbon-free B-SFO sample abruptly deteriorates from roughly 800 to 200 mAh g^−1^ during first 30 cycles, confirming that the carbon substrate does matter to the structural tolerance and thin/stable SEI layer during cycling. It must be accentuated that the reversible capacity and capacity retention of the B-SFO@C electrode significantly outnumber B-SO@C, which is due to the fact that the divergent electroactive potentials of SnO_2_ and Fe_2_O_3_ in the B-SFO@C electrode can permit the Fe_2_O_3_ nanocrystals to not only act as active materials, but also to serve as structural stabilizers [51], and simultaneously enable the amelioration of the reversibility of the conversion reactions of Sn/SnO_2_ in the B-SFO@C electrode to contribute an extra capacity. Specifically speaking, the large work function difference between SnO_2_ and Fe_2_O_3_ can make the lattice of the SnO_2_/Fe_2_O_3_ composite adapt suitably, inducing the construction of a relatively big internal electric field. Such an in-built electric field is able to introduce an additional electromotive force to augment the electronic mobility of the active materials and, finally, strikingly enhance reaction dynamics and ionic/electronic transportation/migration at the interior hetero-interfaces of SnO_2_/Fe_2_O_3_. Actually, TEM and HRTEM have clearly identified the intimately and uniformly packed metal oxide/metal oxide nanoparticles and typical carbon layers. Abundant robust hetero-junctions and -interface boundaries arising from a lattice adaptation between SnO_2_ and Fe_2_O_3_ phase must occur. When such heterostructures come into being between SnO_2_ and Fe_2_O_3_, the electrons will drift to the Fe_2_O_3_ nanoparticles possessing the greater work function from the SnO_2_ (a lower work function), triggering off a positive space charge region on the Fe_2_O_3_ side and a negative space charge region on the SnO_2_ side of the SnO_2_/Fe_3_O_2_ hetero-interfaces. Thus, the built-in electric field takes place around the junction region and the direction is pointed from Fe_2_O_3_ to SnO_2_. This is the identical direction to the electric field of the initial battery, which underlies the gain of the electromotive force of the lithium battery. Furthermore, the long-term high-rate cycling test of the B-SFO@C electrode even at an extremely great current density of 1 and 3 A g^−1^ can still sustain a nearly invariable lithium ion storage behavior, further showing an outstanding high-rate cyclic lifespan (Figure 5d,e). Note that an activation at low current density of 0.2 A g^−1^ was introduced to in advance complete the formation of the unvaried SEI film. The B-SFO@C electrode can afford a reversible capacity of 701 mAh g^−1^ after 500 cycles at 1 A g^−1^ and stores an ultra-stable capacity of 429 mAh g^−1^ even after 1800 cycles at 3 A g^−1^. The electrochemical performances of the B-SFO@C electrode are compared to the SnO_2_-based anode materials published previously (Table S2). It can be unequivocally disclosed that B-SFO@C can ascend into the first echelon of the cutting-edge SnO_2_ based materials, showing that B-SFO@C can vie as the very promising anode material candidate for LIBs. Besides, considering that the (de)alloying reaction that incurs the volume change proceeds mainly below 1 V, the B-SFO@C electrode was purposely measured at 0.2 A g^−1^ in the range of 0.005–1.0 V to trace the morphological variation and the corresponding results (Figure S9) demonstrate the superb cyclic durability with a steady capacity of 343 mAh g^−1^ after 150 cycles. This confirms that capacity fading caused by volume change has been largely alleviated. The electrochemical lithiation must lead the interface boundaries between the Li-enriched Li*_x_*Sn and Sn phases to come into being and resultantly trigger off the stress reinforcement, whilst the gain in the strain energy induces the anisotropic dilation/expansion and abnormal topological evolutions, thus initiating the dramatic mechanical fracture and breakage [30,31,32]. The foregoing events can re-expose the fresh Sn surfaces to electrolytes, thereby resulting in an enhancement in the thickness of the SEI layer upon cycling, and a serious battery performance degeneration. Given that these issues are innately related to stress reinforcement, the endeavor towards the stress regulation approach for Sn-based electrode materials constitutes the paramount footing to enhance a cyclic durability. Seeing that the aggressive volume fluctuation inherently stems from the stress intensification over particle surface layers, such superior cycling stability unambiguously emphasizes that the assembly of the unique sandwich metal–carbon bulk structures with the binary small-sized metal oxides uniformly embedded inside carbon layers can effectively manage and relax the strain energy in the large-volume-variation SnO_2_ anodes for the high-performance LIBs to fulfill the necessary, extraordinary morphological and structural stability requirements (as further supported by the TEM images after cycling in Figure 7). Without the supra mechanism, the material surface will become rather vulnerable to resisting the stress in the lithiation experience, so inducing a limited capacity retention. What’s worse, a high initial discharge capacity and excellent long-term cyclic durability always fall short of being satisfied synchronously thanks to the compromise nexus between the two of them resting with the surface layer thickness. Nevertheless, in spite of such compromise, B-SFO@C can still split the difference to attain a decent cyclability (with 95.1% capacity retention at 100 cycles) at an elevated capacity of 927 mAh g^−1^.

**Figure 6:**
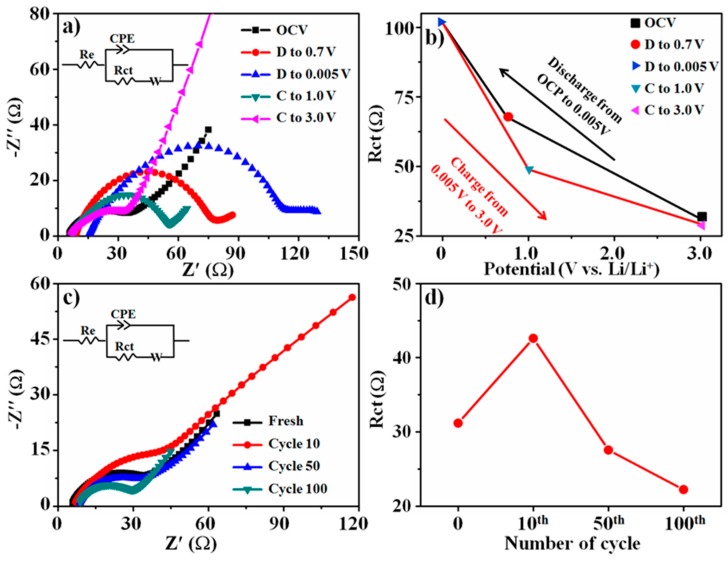
(**a**) The ex situ EIS spectrum evolution of B-SFO@C electrode at the different state of charge and equivalent circuit (inset), (**b**) the corresponding fitted *R_ct_* (**c**) EIS spectra of the B-SFO@C electrode at the different cycles and equivalent circuit (inset), (**d**) the corresponding fitted *R_ct_*.

**Figure 7:**
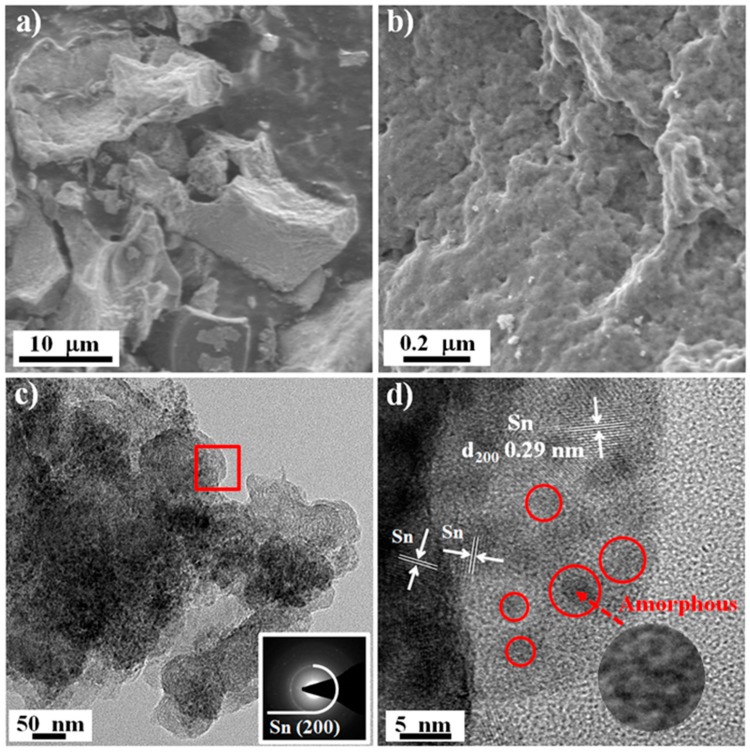
Micromorphology of the cycled B-SFO@C electrode after 500 cycles at 1 A g^−1^: (**a**) Low-resolution and (**b**) High-resolution SEM image, (**c**) Low-resolution TEM image and corresponding SAED pattern (inset), (**d**) HRTEM image.

Electrochemical impedance spectroscopy (EIS) was carried out to further explore reasons for the excellent performance of B-SFO@C. Figure 6a shows the EIS spectra evolution at different states of charge (SOC) in the first cycle, wherein OCV, D to 0.7 V and C to 3.0 V, for example, represent the open circuit voltage, and discharge to 0.7 V and charge to 3.0 V, respectively. All the spectra were well-fitted utilizing equivalent circuits (as described in inset), and comprised of an x-axis intercept at a high frequency, semicircle at high-medium frequencies and the linear line at lower frequencies, which are representative of overall cell resistance (*R_e_*), charge transfer resistance (*R_ct_*) and Warburg impedance, respectively. Combined with the fitted results (Figure 6b), it can be deduced that during the discharge process *R_ct_* gradually increases from 31 to 67 Ω as the potential switches from OCV to 0.7 V, which is due to the formation of poor electronic conductivity of Li_2_O accompanied by reduction of SnO_2_ and Fe_2_O_3_. Then, *R_ct_* substantially increases from 67 to 101 Ω when the potential further dwindles to 0.005 V, which should have the intimate relation with the poor electronic conductivity of SnLi_x_ in situ produced from the alloying reaction. In the subsequent re-charge process, *R_ct_* continuously diminishes back to 29 Ω until the end of charging, which indicates that the electrochemical reaction is highly reversible. The trend of *R_ct_* in the first cycle agrees with prior results [52]. Interestingly, an atypical rise takes place to *R_e_* at 0.005 V (Figure S10), which could relate to the decline in the electrical contacts sparked off due to the dramatic volume expansion [53]. Additionally, the EIS spectra of the B-SFO@C electrode at the different cycles were also depicted in Figure 6c. Similarly, all the spectra also consist of the *x*-axis intercept at the high frequency, half-loop at the high-medium frequencies and the linear line at the lower frequencies. All spectra at the different cycles are very similar except the 10th cycle, implying that over 100 cycles the transfer dynamics can be well retained. Fitted results are provided in Figure 6d, and *R_ct_* is 31 Ω for the fresh B-SFO@C electrode, 48 Ω for the fresh B-SO@C electrode but 82 Ω on the fresh B-SFO electrode (Figure S11). This interprets that the carbon matrix can greatly reinforce the electronic conductivity of B-SFO@C and B-SO@C electrodes, thereby supplying a robust fast electronic connection. The low *R_ct_* in B-SFO@C is responsible for the exceptional rate capability of the B-SFO@C electrode. To be specific, at the 10th cycle, *R_ct_* of the B-SFO@C electrode substantially rises to 42.6 Ω, which can closely correlate with the initial structural rearrangement in first cycles that could arise from the SEI thickening or the formation of the segregated granules [54,55]. Subsequently, the *R_ct_* drops to 27 Ω at the 50th cycle and finally stabilizes at 22 Ω at the 100th cycle, indicating that no appreciable cycling-triggered gain in resistance offers itself. On the one hand, the repeated volume swelling and contraction during cycling make contacts between active materials and carbon matrix closer and closer, which facilitates transfer of electrons from the carbon matrix to the active materials [56,57]. On the other hand, the metallic Sn derived from the irreversible part of conversion reaction of SnO_2_ is a good conductor and acts as conductive matrix, which further reduce the *R_ct_* [58]. As the SEI layer thickening can augment resistance, the *R_ct_* evolution combined with post-characterization (Figure 7) can reveal that the excellent structural resilience/flexibility of B-SFO@C to maintain textural stability can aid in yielding the stable and thin SEI layer and keeping small resistance in spite of fierce electrochemical events.

To further disclose the structural elasticity/robustness, the micro-morphology of B-SFO@C subjected to 500 cycles at 1 A g^−1^ was further examined by SEM and TEM. As shown in Figure 7a, the cycled B-SFO@C electrode can still preserve a micron-sized construction well, without any cracks on its surface even if the surface is somewhat distinguished from that of the fresh B-SFO@C electrode. After cycling, the surface becomes smoother and denser, owing to SEI formation. The magnified SEM image (Figure 7b) indicates no obviously spotted fracturing, aggregation, or pulverization on the electrode surface, which verifies the excellent morphologic stability. Such phenomena ensue from a symmetric and uniform volume variation of the metal phases, further elucidating that the shortcomings of Sn-based materials seem to be fully overcome by a straightforward structuring (namely, embedment of the binary small-sized metal particles into the carbon lamella of a compact monolithic geometry). The microstructural integrity can further be confirmed by TEM image (Figure 7c), where the metal-in-carbon sandwich structure of constituent particles is retained. That is, the metal nanoparticles are observed to be still well dispersed and intercalated inside carbon layers with no obvious carbon aggregation/fracturing taking place, very similar to the structural motifs revealed by Figure 3c,d. The size of metal nano-particles is very close to the case in the fresh one, suggesting that the metal particle coarsening can be powerfully inhibited and that the unique intercalation layout is able to strongly protect the metal particles from cracking due to the nonuniform and huge volume evolution over initial cycles. These effects can make sure that the morphologic evolutions will not become increasingly serious over the cycling to circumvent the fracturing/pulverization, thus avoiding both the breakage of the preformed SEI layer and endless SEI amassment upon repeated cycles. Corresponding SAED pattern displays the diffraction rings of Sn; however, no diffraction rings characterizing SnO_2_ and Fe_2_O_3_ are discerned. Nevertheless, the HRTEM image (Figure 7d) reveals that there are plentiful ultrafine amorphous nanoparticles being detected (noted by red circle) except for the crystalline Sn. On the grounds of the preceding reports [59,60], these ultrafine amorphous nanoparticles may be SnO_2_ and Fe_2_O_3_—which accounts for the absence of diffraction rings of SnO_2_ and Fe_2_O_3_ in SAED pattern. Coupling the electron spectroscopy with the cyclic behavior, the characteristics of the surface and morphological integrity can become manifest in the production of steady and thin SEI layers, bringing forth the improved cyclic performance. In short, in conjunction with the excellent cyclic longevity and rate handling, the morphologic characterization before and after the prolonged cycling can undeniably disclose an extraordinary shape memory effect for the topologically frustrated lattice reorganization during the reduplicative (de)lithiation chemistry, leading the electrode to survive even over long-term cycling, i.e., the intercalation structure permits metals to dilate and constringe without disintegration during cycling. As discussed above, in essence, the transcendent structural and morphologic resilience arises from effective stress management from binary metal nanoparticles-in-carbon structures. Accordingly, we are convinced that although it features the micro-sized dimensionality the binary metal-in-carbon architecture not only acts as a stress relaxation regulator to mitigate the compressive stress of lithiated metal phase but also retains the morphological integrity without any mechanical failure by dwindling the tensile stress intensified to carbon layers, inducing the low electrode expansion effect.

## 4. Conclusions

To sum up, impressive lithium ion storage properties with exceptional power/energy ratings and cyclic durability have been realised without sophisticated nanostructuring/nanoscaling, but rather only through architecting the binary metal oxide-intercalated carbon bulk configuration. To be precise, using an eco-benign, expeditious, and economical gel pyrolysis recipe, a high density of homogeneous SnO_2_ and Fe_2_O_3_ nanoparticles were successfully inlayed into the carbon lamellas in a conductive protecting carbon sheath. A facile low-temperature annealing imparts the highly dispersed-SnO_2_ and Fe_2_O_3_ nanoparticles with an average size of about 5 nm, as well as the exceptional anti-coarsening and surface-to-volume atom ratio. It is this novel anode material that potently adopts this distinctive microscopic sandwich superstructure motif to endow the topologically frustrated host structure for the tenable conversion reaction with easy and defect-resistant Li motion and multielectron events. The “carbon sheath” intrinsic matrix of the nanohybrid combining with the “Li_2_O” formed from the conversion reaction can function as a mixture buffering matrix that conduces to keep the electro-chemically formed nanoscaled metal particles apart and thwart their agglomeration during the Li-Sn alloy formation/decomposition, thus curbing the volume/stress side-effect and capacity fading by maintaining the electrode integrity. Factoring in the low cost of raw materials, B-SFO@C can be revered as an eminent anode substance for stationary LIBs. The employed methodology here can recapitulate a very appealing arena towards maturing the metal-carbon nanohybrid wherein both distinguished virtues of the large theoretical capacity of metals and exceptional conducting/buffering functions of carbon are able to be sufficiently leveraged, complemented and finally rolled into one. A distinctive engineering tenet or direction for anode substances within lithium-ion cells can be inferred from the insights into this superior electrochemical reaction performance, coupled with the rich chemistry of synthetic strategies. That is, maintaining the high dispersion state of the ultrafine multi-component metal oxide nanoparticles within the micro-sized, compactly packed, and elastic carbon nanostructure is vital to improve the reversibility, cyclability, capacity, and rating of the conversion reaction-based metal oxide electrodes. In the case of the fruitful anode material engineering, we can envisage that an extensive portfolio of the multi-nary metal oxides (using the IVA, VA, and VlllB metals such Sb, Bi, Se, Ge with the greater capacities) and micron dimensional compact carbon matrix turns feasible, and the miscellaneous conversion reaction nanocomposite anode materials can be innovated with a fine-modulating of their Li storage behaviours. As long as the metal oxides encompass an available redox-active ingredient, the carbon substrates may get enlisted in both electronic conductivity and textural stability without respect to crystalline structures and combinations. In addition, because conversion reactions of different metal oxides can readily occur in varied voltage windows, a built-in electronic field between different and adjacent metal nanocrystals comes into being, and so the electrochemical process can smoothly take place to further circumvent a drastically aggressive volume perturbation, dendritic plating behaviour, and stress intensification, in sharp contrast with a unitary metal oxide electrode. Therefore, the selection of the sequential (de)lithiation potentials of the combined metal components can also serve as an additional paramount rudder for manipulating the electrochemical properties of the hybrid material electrodes. All taken together, the multi-nary metal oxide–carbon composite with the metal nanoparticles uniformly sequestrated into the lamellar carbon matrix could inaugurate an alternative roadmap for both academic research and the realistically-related deployment of greatly durable, high-rate, conversion-type anode substances in all batteries technologies.

## Supplementary Materials

The following are available online at https://www.mdpi.com/2079-4991/10/2/249/s1, Figure S1: XPS spectra of C 1s for B-SFO@C sample; Figure S2: TGA curves of the B-SFO@C sample; Figure S3: Raman spectrum of the B-SFO@C sample; Figure S4: EDX mapping images of the B-SFO@C sample; Figure S5: Nitrogen adsorption–desorption isotherms of B-SFO@C sample; Figure S6: (a) SEM images of B-SO@C sample, (b) Low-resolution and (c) High-resolution SEM image of B-SFO sample; Figure S7: Magnified TEM image of the B-SFO@C sample; Figure S8: Cyclic performance of carbon matrix at 0.2 A g^−1^ in the range of 0.005–3.0 V; Figure S9: Cyclic performance of B-SFO@C electrode at 0.2 A g^−1^ in the range of 0.005–1.0 V; Figure S10: the corresponding fitted *R_e_* at different SOC; Figure S11: EIS spectra of fresh B-SO@C and B-SFO electrodes the corresponding fitted *R_ct_* (inset); Table S1: The weight fractions of SnO_2_ and Fe_2_O_3_ in B-SFO@C sample calculated different methods; Table S2: The electrochemical performances of B-SFO@C and SnO_2_-based composites anode materials in the previous reports.
