# Supplementary material for: Bulk-Like SnO2-Fe2O3@Carbon Composite as a High-Performance Anode for Lithium Ion Batteries
Source: Nanomaterials (Basel). 2020 Jan 30;10(2):249. doi: 10.3390/nano10020249 (PMC7075132; doi:10.3390/nano10020249)
Supplement: Supplementary file 1 [file nanomaterials-10-00249-s001.pdf]

Supplementary Materials: Bulk-Like SnO_2-Fe_2O_3@Carbon Composite as a High-Performance Anode for Lithium Ion Batteries

Jie Deng [1,†], Yu Dai [2,†], Zhe Xiao [3], Shuang Song [2], Hui Dai [2,4], Luming Li [1,5,*] and Jing Li [2,*]

[1] College of Pharmacy and Biological Engineering, Chengdu University, Chengdu 610106, China; dengjie@cdu.edu.cn

[2] Department of Chemical Engineering, Sichuan University, Chengdu 610065, China; daiyuscu@163.com (Y.D.); 2016323050027@stu.scu.edu.cn (S.S.); daihui18@cdut.edu.cn (H.D.)

[3] Institute of New Energy and Low Carbon Technology, Sichuan University, Chengdu 610207, China; 2017226220007@stu.scu.edu.cn

[4] College of Materials and Chemistry & Chemical Engineering, Chengdu University of Technology, Chengdu, 610065, China;

[5] Institute of Advanced Study, Chengdu University, Chengdu 610106, China

* Correspondence: liluming@cdu.edu.cn (L.L.); jingli0726@g.ucla.edu (J.L.)

† These authors contributed equally to this work.

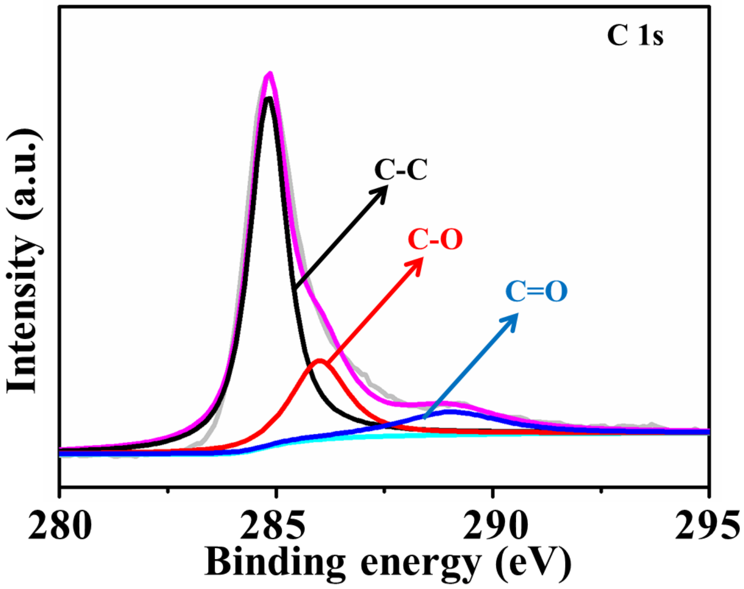

Figure S1. XPS spectra of C 1s for B-SFO@C sample.

Table S1. The weight fractions of SnO_2 and Fe_2O_3 in B-SFO@C sample calculated different methods

methods	SnO_2	Fe_2O_3
ICP	63.5%	9.2%
XPS	47.2%	7.5%

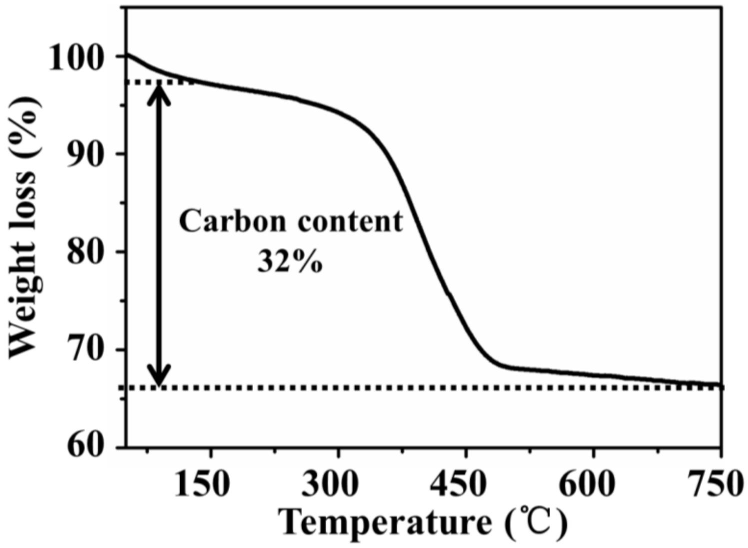

Figure S2. TGA curves of the B-SFO@C sample.

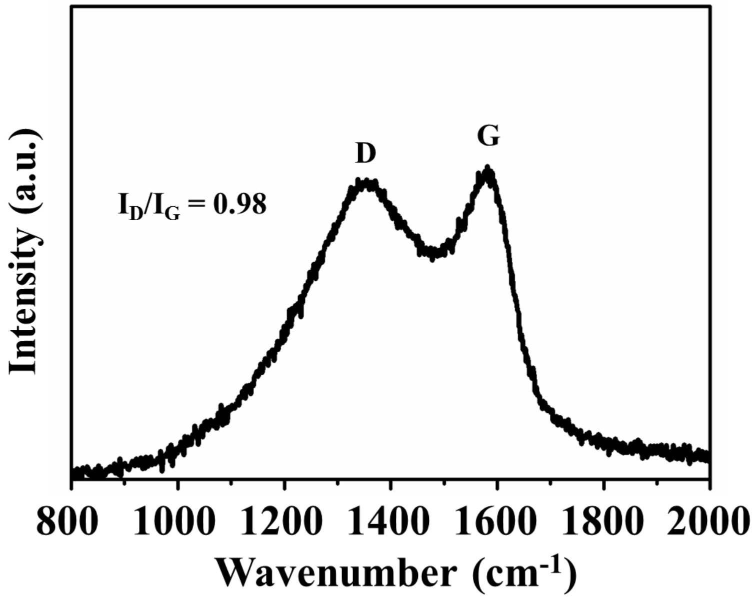

Figure S3. Raman spectrum of the B-SFO@C sample.

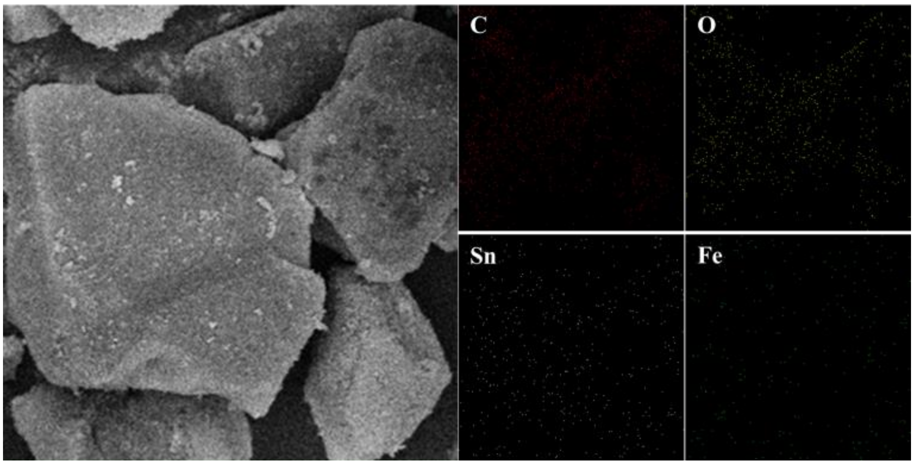

Figure S4. EDX mapping images of the B-SFO@C sample.

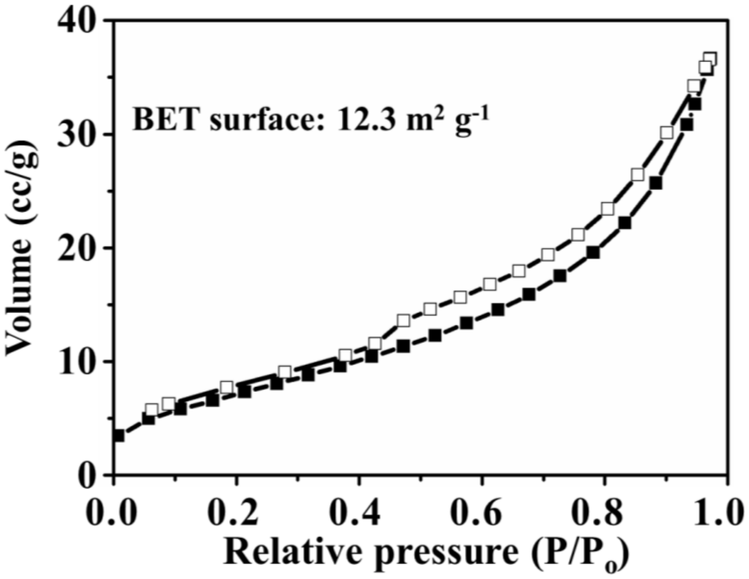

Figure S5. Nitrogen adsorption–desorption isotherms of B-SFO@C sample.

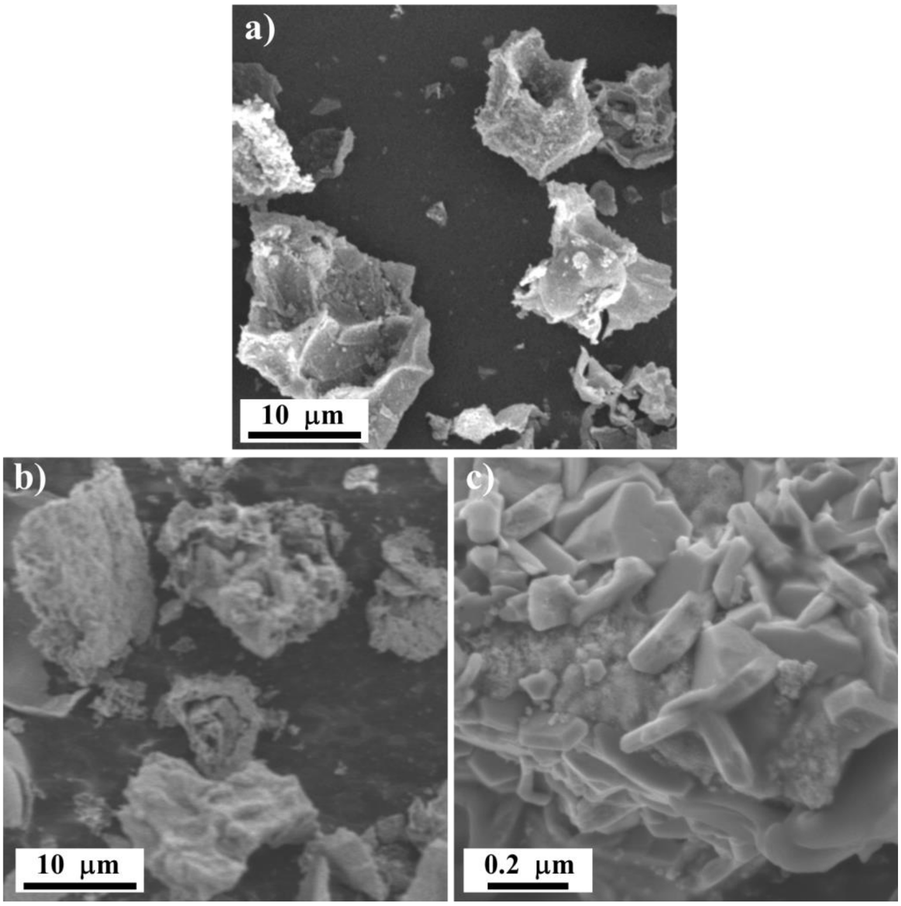

Figure S6. (**a**) SEM images of B-SO@C sample, (**b**) Low-resolution and (**c**) High-resolution SEM image of B-SFO sample.

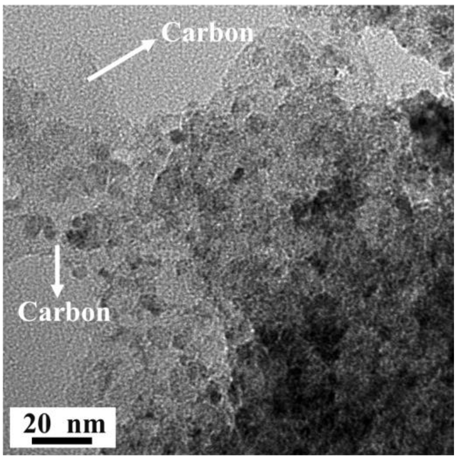

Figure S7. Magnified TEM image of the B-SFO@C sample.

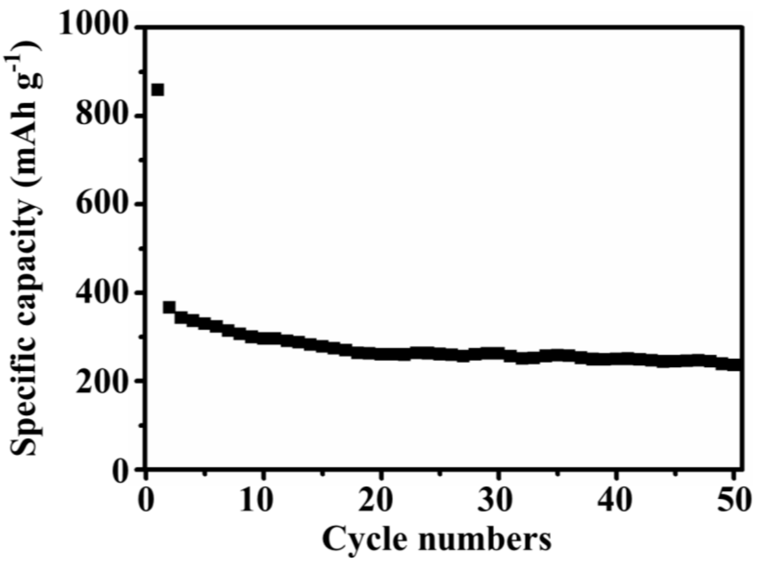

Figure S8. Cyclic performance of carbon matrix at 0.2 A g^{-1} in the range of 0.005–3.0 V.

Table S2. The electrochemical performances of B-SFO@C and SnO_2-based composites anode materials in the previous reports.

Sample	Current density (A g^{-1})	Initial coulombic efficiency	Remained Capacity (mAh g^{-1})	Cycle number	Ref.
Bulk SnO_x@C	0.2<br>1	46.3%	885.8<br>637.2	360<br>1000	1
Carbon-Encapsulated Porous SnO_2	0.05	41.6%	870.9	120	2
Honeycomb-like SnO_2@C	0.2<br>1	66.2%	940<br>400	150<br>500	3
SnO_2@CNT	0.2<br>1	62.5%	546<br>398	100<br>150	4
3D h-SnO_2-Fe_2O_3@RGO	0.2	61.3%	830	100	5
rGO/ Fe_2O_3/ SnO_2	0.4	63%	700	100	6
Fe_2O_3@SnO_2/GS	0.1	60.8%	1015	200	7
SnO_2/Fe_2O_3/RGO	0.2<br>1	46%	795<br>690	220<br>1000	8
SnO_2-Fe_2O_3/SWCNTs	0.2<br>1	64.9%	692<br>553	50<br>100	9
B-SFO@C	0.2<br>1<br>3	70%	927<br>701<br>429	100<br>500<br>1800	This work

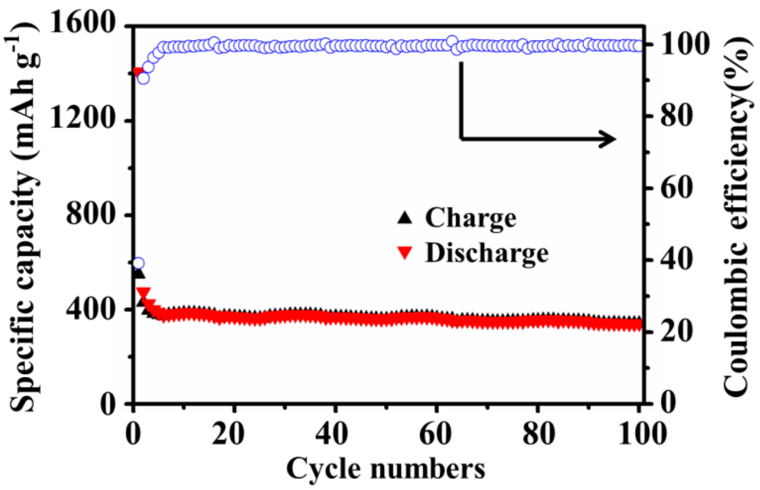

Figure S9. Cyclic performance of B-SFO@C electrode at 0.2 A g^{-1} in the range of 0.005–1.0 V.

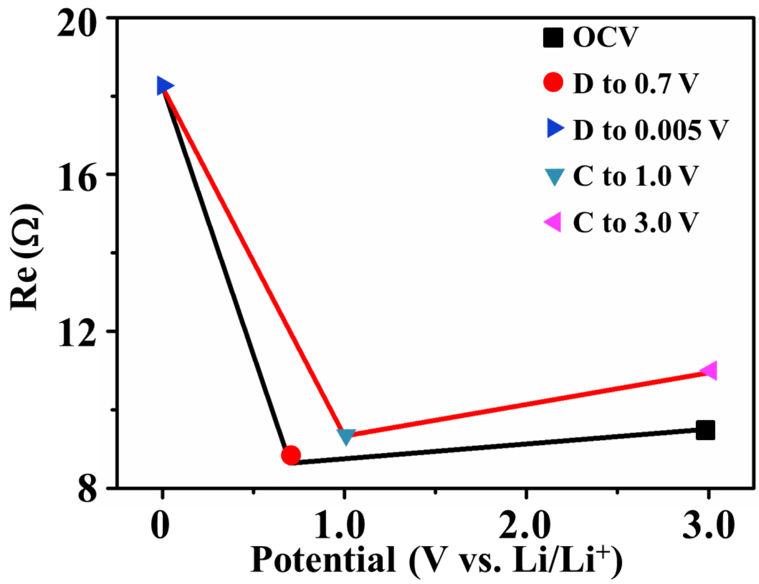

Figure S10. The corresponding fitted R_e at different SOC.

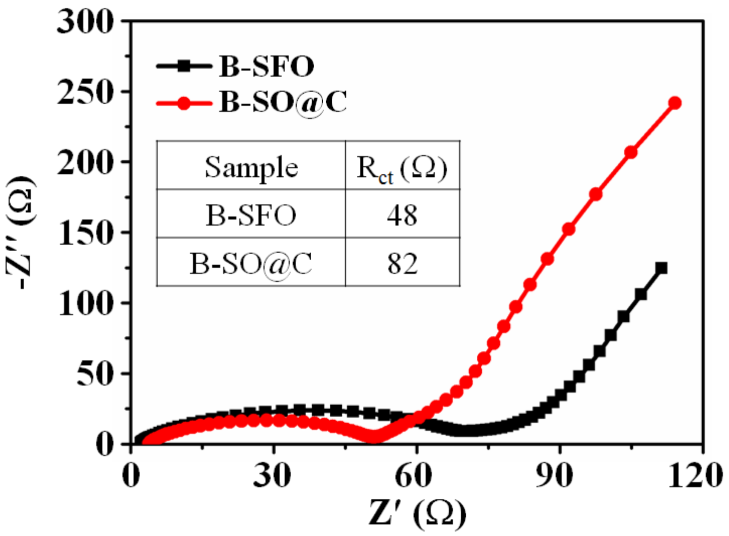

Figure S11. EIS spectra of fresh B-SO@C and B-SFO electrodes the corresponding fitted R_{ct} (inset).

References

1 Tian, Q.H.; Hong, Z.M.; Chen, P.; Zhang, Z. X.; Li, Y. Bulk SnO_x@C composite for improved lithium storage. *J. Alloy. Compd.*, **2018**, *740*, 312–320.

2 Huang, B.; Li, X.H.; Pei, Y.; Li, S.; Cao, X.; Masse, R.C., Cao, G.Z. Novel carbon-encapsulated porous SnO_2 anode for lithium-ion batteries with much improved cyclic stability. *Small*, **2016**, *12*, 1945–1955.

3 Wang, H.K.; Wang, J.K.; Cao, D.X.; Gu, H.Y.; Li, B.B.; Lu, X.; Han, X.G.; Rogach, A.L.; Niu, C.M. Honeycomb-like carbon nanoflakes as a host for SnO_2 nanoparticles allowing enhanced lithium storage performance. *J. Mater. Chem. A*, **2017**, *5*, 6817–6824.

4 Cheng, Y.Y.; Huang, J.F.; Qi, H.; Cao, L.Y.; Yang, J.; Xi, Q.; Luo, X.M.; Yanagisawa, K.; Li, J.Y. Adjusting the chemical bonding of SnO_2@CNT composite for enhanced conversion reaction kinetics. *Small*, **2017**, *13*, 1700656–1700666.

5 Zhao, B.; Xu, Y.T.; Huang, S.Y.; Zhang, K.; Yuen, M.M.F.; Xu, J.B.; Fu, X.Z.; Sun, R.; Wong, C.P. 3D RGO frameworks wrapped hollow spherical SnO_2-Fe_2O_3 mesoporous nano-shells: fabrication, characterization and lithium storage properties. *Electrochim. Acta*, **2016**, *202*, 186–196.

6 Xia, G.F.; Li, N.; Li, D.Y.; Liu, R.Q.; Wang, C.; Li, Q.; Lu, X.J.; Spendelow, J.S.; Zhang, J.L.; Wu, G. Graphene/Fe_2O_3/SnO_2 ternary nanocomposites as a high-performance anode for lithium ion batteries. *ACS Appl. Mater. Interfaces*, **2013**, *5*, 8607–8614.

7 Liu, S.; Wang, R.H.; Liu, M.M.; Luo, J.Q.; Jin, X.H.; Sun, J.; Gao, L. Fe_2O_3@SnO_2 nanoparticle decorated graphene flexible films as high-performance anode materials for lithium-ion batteries *J. Mater. Chem. A*, **2014**, *2*, 4598–4604.

8 Lee, K.; Shin, S.; Degen, T.; Lee, W.; Yoon, Y.S. In situ analysis of SnO_2/Fe_2O_3/RGO to unravel the structural collapse mechanism and enhanced electrical conductivity for lithium-ion batteries. *Nano Energy*, **2017**, *32*, 397–407.

9 Wu, W.L.; Zhao, Y.; Li, J.X.; Wu, C.X.; Guan, L.H. A ternary phased SnO_2-Fe_2O_3/SWCNTs nanocomposite as a high-performance anode material for lithium ion batteries. *J. Energy Chem.*, **2014**, *23*, 376–382.
